# Brainstem tumors in children: a monocentric series in the light of genetic and bio-molecular progress in pediatric neuro-oncology

**DOI:** 10.3389/fped.2023.1193474

**Published:** 2023-10-23

**Authors:** Rel Gerald Boukaka, Pierre-Aurélien Beuriat, Federico Di Rocco, Alexandre Vasiljevic, Alexandru Szathmari, Carmine Mottolese

**Affiliations:** ^1^ Department of Pediatric Neurosurgery, Hôpital Femme Mère Enfant, Hospices Civils de, Lyon, France; ^2^ Université Claude Bernard, Lyon 1, Lyon, France; ^3^ Department of Pathology and Neuropathology, Groupement Hospitalier Est, Hospices Civils de Lyon, Lyon, France

**Keywords:** pediatric, brainstem, dipg, benign, surgery

## Abstract

**Introduction:**

Brainstem tumors represent a challenge. Their management and prognosis vary according to anatomopathological findings and genetic and bio-molecular fingerprints. We present our experience with pediatric brainstem tumors.

**Material and methods:**

All patients admitted for a brainstem tumor at the Pediatric Neurosurgical Unit at Hôpital Femme Mère Enfant hospital between January 1997 and December 2019 were considered. Patients data were obtained through a retrospective review of the medical records; follow-up was from the last outpatient consultation.

**Results:**

One hundred and twelve patients were included. Eighty-five patients (75.9%) had open surgery or stereotactic biopsy. Thirty-five patients were treated for hydrocephalus. Sixty-six received an adjuvant treatment. Several protocols were adopted according to the SFOP and SIOP during this time period. The overall survival rate was 45% with a median follow-up of five years (range 1–18 year). However, the survival rate was very different between the diffuse intrinsic pontine gliomas (DIPG) and the others tumor types. If we exclude the DIPG (59 patients), of which only 1 was alive at 3 years, the survival rate was 90.6% (only 5 deaths over 53 patients) with a median follow up of 5 years.

**Conclusions:**

Our series confirms that benign tumors of the brainstem have a good survival when treated with surgical removal ± adjuvant therapy. Diffuse pontine gliomas continue to have a dismal prognosis. Individualized treatment based on molecular fingerprints may help to select the best adjuvant therapy and hence potentially improve survival.

## Introduction

Brainstem tumors represent 15% of children brain tumors; 80% of children brainstem tumors are diffuse intrinsic pontine gliomas (DIPG) that have a dismal prognosis. Brainstem tumors were first described by Kummel in 1881 (1) and Monakow (2). The first nosological classification was reported in 1926 by Bailey and Cushing who emphasized, for the first time, that brainstem gliomas could develop from certain embryological cells (3). The dismal history of brainstem tumors and their catastrophic evolution was already noted in their report.

In 1989, a report of the French Speaking Society of Neurosurgery on Brainstem Tumors showed the progress achieved in term of radiological diagnosis using MRI, histological classification and genetic knowledge. The role of surgery to treat exophytic lesion of the mesencephalon, of the bulbo-medullary region or focal lesions anywhere in the brainstem was also recognized. However, the survival rate of diffuse intrinsic gliomas did not improve (4). The purpose of this paper is to share our experience with a consecutive series of 112 children with brainstem tumors treated from 1997 to 2019 and to emphasize the role of recent genetic and bio-molecular progress.

## Material and methods

All patients who were admitted and diagnosed with a brainstem tumor in the Pediatric Neurosurgical Unit at the “Hôpital Femme Mère Enfant” (Hospices Civils de Lyon, Lyon, France) between January 1997 and December 2019 were included in this study. Patient data were obtained through a retrospective review of the medical records from our data base; follow-up data was from the last outpatient visit. All patients without a clinical history or radiological images and without a documented post-surgical follow-up were excluded from this study.

The study protocol was approved by the local ethics committee.

One hundred and twelve patients were included in this study. All patients were diagnosed with a cranio-medullary MRI with and without gadolinium. The male/female ratio was 0.94 and the age varied from 6 months to 18 years with a median age of 8.9 years. The symptomatology that led to the diagnosis was unknown in 44 patients (39.3%), progressive in 63 patients (56.2%), acute in 2 patients (1.8%) and incidental in 3 patients (2.7%). At diagnosis, 14% of patients presented a deficit of the mixed cranial nerves, 21% cerebellar troubles with balance disorders, 16% nystagmus and torticollis, 10% impaired consciousness while 38% presented with a mild intracranial hypertension. According to the classification of Choux (5), 59 patients were classified as belonging to the group I (52.7%), 25 to the group II (22.3%), 26 to group III (23.2%), and 2 to group IV (1.8%).

Table 1 shows the different tumor locations. The surgical removal was definite as Growth total Resection (GTR), when the post-operative MRI showed a total removal, as Near Total resection (NTR) when the removal was at least of 90% of the pre-operative volume, and subtotal removal (STR) when less of 90% of the tumor was removed (6).

**Table 1:** Tumors localisation.

Localization	*N* = 112 (100%)
Pons	59 (53%)
Tectal region	15 (12.5%)
Bulbo-medullary region	16 (14.3)%
Latero-bulbar region	8 (7.1%)
Ponto-cerebellar angle	4 (3.6%)
Bulb	3 (2.6%)
Ponto-bulbar region	6 (5.3%)

**Figure 1:**
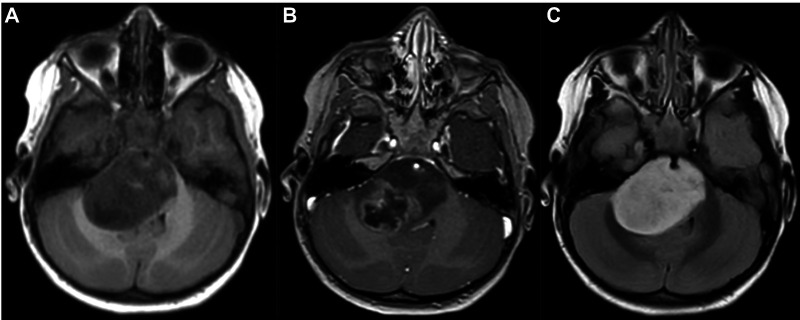
MRI scan in an axial 3D T1-weighted contrast (**A**), 3D T1-weighted contrast after gadolinium injection (**B**) and 3D T2 Flair-weighted contrast (**C**) showing a typical diffuse intrinsic pontine glioma (DIPG).

Out of the 34 patients with DIPG the histological diagnosis were a grade IV diffuse malignant glioma in 24 patients, a grade III astrocytary glioma in 3 patients, an anaplastic gliomas in one patient, a grade III oligo-astrocytary glioma in one patient, an AT/RT in two patients and an ETANTR in one patient. The radiological findings were not different between the different tumor types (Figure 1).

**Figure 2:**
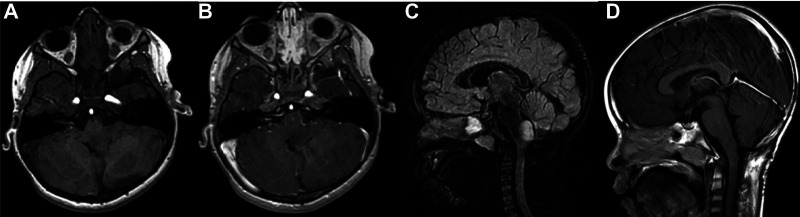
Pre operative MRI scan in an axial 3D T1-weighted contrast (**A**), 3D T1-weighted contrast after gadolinium injection (**B**) and sagital 3D T2 Flair-weighted contrast (**C**) showing a typical typical exophytic bulbo-medullar ganglioglioma. D is the post operative control in a sagital 3D T1-weighted contrast after gadolinium injection showing a complete removal.

**Figure 3:**
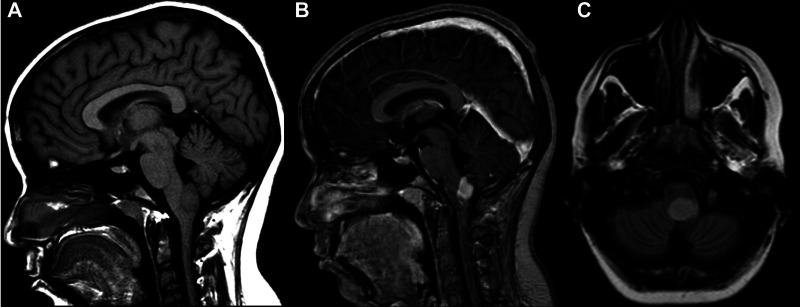
MRI scan in a sagital 3D T1-weighted contrast (**A**), 3D T1-weighted contrast after gadolinium injection (**B**) and 3D T2 Flair-weighted contrast (**C**) showing a typical typical exophytic bulbo-medullar ganglioglioma.

Histology of focal and exophytic brainstem tumors were a grade I pilocytic astrocytoma (Figure 2) in 63% of cases of, a ganglioglioma (Figure 3) in 29% and a oligo-astrocytary tumors in 8% of cases.

## Results

Eighty-five patients (75.9%) had open surgery or biopsy (open or stereotactic). For DIPGs, the biopsy was proposed to the family but in 27 patients the biopsy was refused so the diagnosis was based on neuroradiological criteria. Table 2 details the histopathological findings. Forty-four of the surgical procedures were biopsies either open (23 patients), frame-based (16 patients) or frameless (5 patients). The other surgical procedures were a direct surgical approach aimed at tumor removal. Three patients were operated for a recurrence and no patient received a third surgical procedure. One patient was re-operated for a new biopsy because the first procedure did not permit a diagnosis. Forty-two patients had surgery for tumor removal with a total surgical resection in 20 cases (47.6%), a subtotal resection in 13 patients (30%), and a partial resection in 9 patients (22.4%). Thirty-five patients were treated for hydrocephalus during forty-eight surgical procedures. Thirty of these procedures were ventriculo-peritoneal (VP) shunt while 18 endoscopic third ventriculocisternostomy (ETV). Hydrocephalus needed to be treated after surgery in 12 patients. Four patients needed VP shunt after surgery despite a previous ETV and three patients had new ETV after the direct approach on the tumor.

**Table 2:** Anatomopathological findings.

Anatomopathological findings	*N* = 85 (100%)
Pilocytic Astrocytoma	31
Grade IV astrocytoma	26
Ganglioglioma	8
Grade III astrocytoma	7
Gangliocytoma	5
Grade II astrocytoma	3
AT/RT	2
ETANTR	1
PNET	1
Malignant glioneuronal tumor	1

ETANTR, Embryonal tumor with abundant neuropil and true rosettes; AT/RT, Atypical Teratoid Rhaboid Tumor; PNET, primitive neuroectodermal tumor.

We tabulated the post-operative complications separately for patients who underwent biopsy and for those who underwent tumor removal. In the biopsy group, 29% of the patients (14/48) had postoperative complications. One patient died after a diffuse edematous reaction of the brainstem, 2 patients presented a reversible bradycardia, one patient had respiratory troubles that regressed after the administration of corticoids, 2 patients had worsening of the pre-operative cerebellar syndrome, 3 patients had worsening of the pre-operative motor deficit (complete deficit in one case), 5 patients presented new cranial nerves deficit (3 facial nerve palsy and 2 swallowing problems). 29% (12/41) of patients who had surgery aimed at tumor removal had postoperative complications: 12 had a new cranial nerve deficit (7 swallowing troubles, 2 VI^th^ nerve palsy, 3 peripheral facial nerve palsy), 2 patients presented a Parinaud syndrome, 2 patients had worsening of the pre-operative cerebellar syndrome, one patient developed a left hemiparesis and 2 patients developed tetraparesis with respiratory troubles. In one case a small intratumoral hemorrhage was visualized without clinical symptoms, 2 patients developed an epidural hematoma that didn't need a surgical treatment, and two patients developed a pseudo-meningocele treated with furosemide.

Five patients with a DIPG without histone mutation presented a metastatic evolution in spite of two different chemoterapic treatment.

### Molecular genetic characteristic

We obtained complete histopathological and biomolecular analysis in 40 patients out of the 85 operated patients.

In patients with a diffuse gliomas, a mutation K27M of the histone H3.3 (H3K27M) was found in 18 cases. This mutation is characterized by the substitution of a lysine (K) with the methionin (M). It was absent in 10 cases. Amplification of PDGFRA was seen in two cases.

Among 15 tested circumscribed benign gliomas, the presence of BRAF molecular alterations was recorded in 9 cases. BRAF alterations included KIAA1549-BRAF fusion that is more specific of pilocytic astrocytomas (5 cases) and the BRAF V600E mutation that is more frequent in ganglioglioma (4 cases).

Two patients were diagnosed as AT/RT. One patient had an embryonal tumor with multi-layered rosettes (ETMR) with an ETANTR phenotype.

We were able to define five groups of patients (Table 3): diffuse gliomas with H3K27M mutation (18 patients), diffuse gliomas without H3K27M mutation (10 patients), circumscribed benign focal gliomas with BRAF alterations (9 patients), and embryonal tumors (3 patients). The presence of the H3K27M mutation allowed the inclusion of patients with DIPG in specific therapeutic protocols such as BIOMEDE. The presence of BRAF-V600E mutation and KIAA1549-BRAF fusion favoured the use of targeted therapy in patients with partial removal or recurrence.

**Table 3:** Summary of the molecular findings.

Radiology/histopathology	Diffuse glioma		Circumscribed/exophytic glioma		Embryonal tumors	No detected BRAF alterations
Molecular data	H3K27M+	H3K27M−	BRAF V600E	KIAA1549-BRAF fusion	AT/RT, ETANTR	No detected BRAF alterations
Number of patients	18	10	4	5	3	6

### Complementary treatment

Sixty-six patients (59%), had postoperative chemotherapy according to the indications of our neuro oncological team and according to the protocol of the SFOP. Several types of protocol were used (BIOMEDE, LGG, TRONC 98, TARCEVA, CILENT, STUPP, BB SFOP, VELBE, PNET HR) following recommendations of the local neuro-oncological team. All patient with DIPGs received a complementary treatment.

Table 4 shows the different protocols used, and the survival at one, two and five years. Twenty-six patients were treated with only chemotherapy at beginning and 31 patients were treated with chemotherapy followed by radiotherapy. Three patients received chemotherapy after a new surgical procedure for tumor recurrence. Six more patients received chemotherapy for tumor progression on the follow-up MRI scan. Five patients with a DIPG received only radiotherapy due to rapid progressing neurological deterioration.

**Table 4:** Summary of all treatment protocol used for high grade tumor with the corresponding survival rate.

Protocol	Number of patients	1-year survival (%)	2-year survival (%)	3-year survival (%)
BIOMEDE	16	64	6.1	6.1
TRONC 98	15	53	20	0
CILENT	3	33	0	0
TARCEVA	4	0	0	0
TEMODAL	4	0	0	0
PNET HR	2	0	0	0

### Survival

At last follow-up, the overall survival rate was 45% with a median follow-up of 7.5 years (range from 1 to 23 years). However, this survival rate was very different between DIPG and circumscribed/exophytic gliomas. For DIPG, the median survival is inferior to one year (10.7 months) with a range between 4 months and 36 months. The DIPG with a survival of 36 months was H3.1 mutated with the lost of PTEN. If we exclude the 59 DIPG (with only 1 patient alive after 3 years of follow-up), the survival rate of the non-DIPG tumors of our cohort was 90.6% (only 5 deaths over 53 patients) showing that benign tumors of the brainstem can have a good survival. The median survival for all patients with benign focalized or exophytic brainstem tumors was 13 years with a range varying from two to 23 years. Eleven patients had a survival superior to fifteen years with a range between 15 and 23 years.

**Figure 4:**
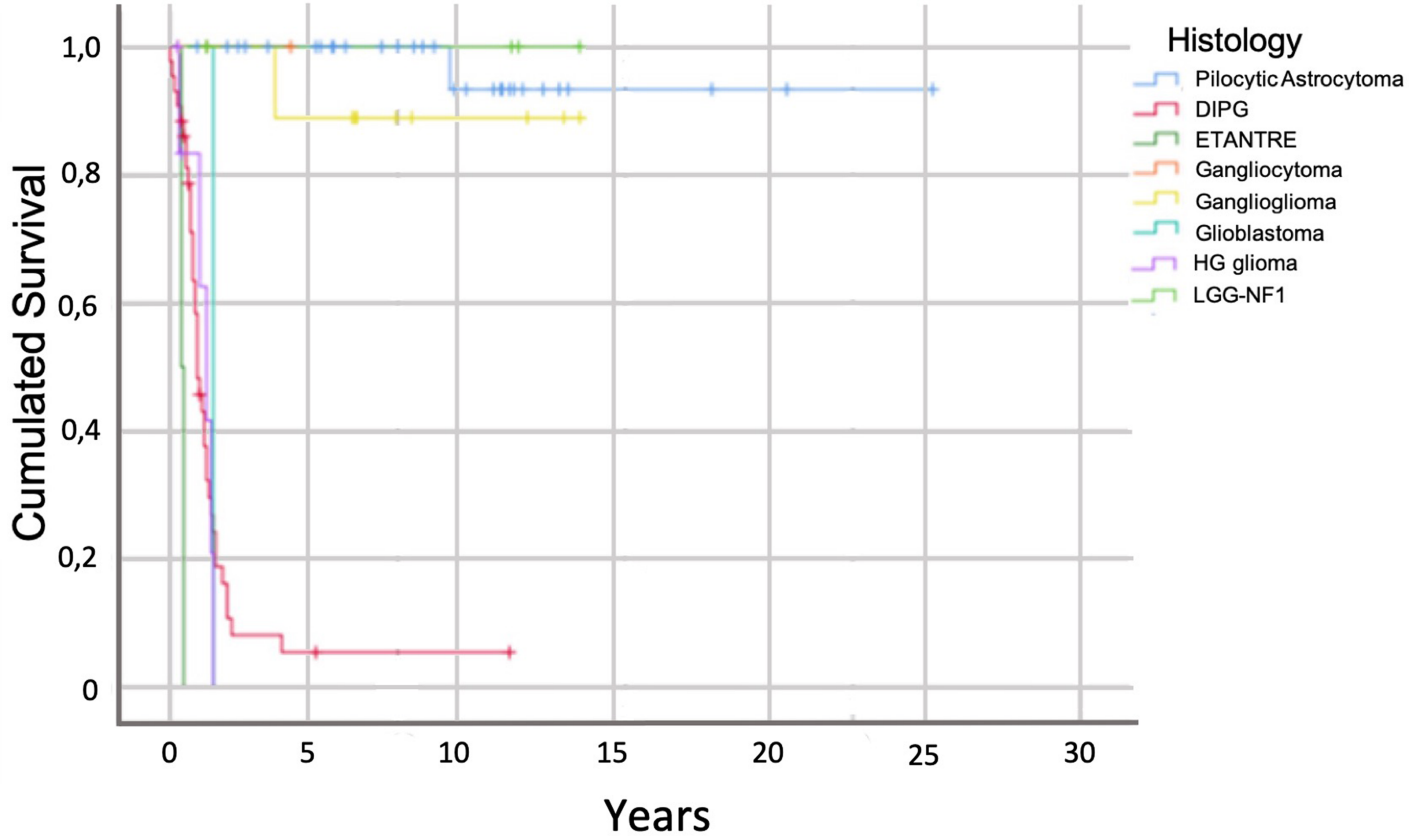
Survival analysis of the different histology types.

The mortality was 14.3% (*n* = 2) for the tectal plate tumors (Grade IV astrocytoma and 1 patient not operated), 6.25% (*n* = 1) for the bulbo-medullary region tumors (ganglioglioma), 0% for the ponto-bulbar and ponto-mesencephalic region exophytic tumors, 9.1% (*n* = 1) for the latero-bulbar and bulbar region tumors and 25% (*n* = 2) for the ponto-cerebellar tumors (AT/RT). Figure 4 shows the survival curve of all the brainstem tumors highlighting the different evolution between the DIPG and the non-DIPG tumors.

## Discussion

Brainstem tumors still represent a great challenge for paediatric neurosurgeons and neuro- oncologists because the survival rate remains low despite the recent progress in genetic and molecular knowledge (7–9). The balance is always between the lesions accessible to a surgical removal and the more infiltrating lesions such as DIPGs that are characterized by a dismal prognosis. Brainstem tumors were already described in the 50s and a French monograph reported the first modern classification and description of these insidious lesions (10). After the first report of Kummel in the US in 1881 (1), Monakow (2), Bailey and Cushing (3) reported the first modern description of this pathology in 1926 and stated that it was impossible to treat these tumors. Moreover, Matson, the father of the modern pediatric neurosurgery, stated that surgery was not indicated for treatment of this tumors (11). Brainstem tumors represent 15% of tumors of the central nervous system in children and 80% are located in the pontine region. There is no preference of sex and the mean age at diagnosis is between 7 and 9 years. In 1989, Guy, Jan and Guegan pointed out the progress in the field of radiology, electrophysiology and in the surgical removal of exophytic brainstem tumors (4). This report confirmed the dismal outcome in terms of clinical results and survival for DIPG in comparison with circumscribed or exophytic brainstem tumors that are generally histologically more benign and accessible to large surgical resection (4).

Stroink (1986) classified brainstem lesion in 4 differents groups: exophytic lesions (group I), intrinsic lesions without enhancement (group II a), intrinsic lesions with lateral or ventral extension (group II b), intrinsic lesion with enhancement (group III); focal lesions (group IV) (5). We have used the classification of Choux (12) that separates the lesion in diffuse, intrinsic focal, extrinsic focal, and cervico-medullary tumors. This classification was used in because it is a French classification. The diffuse forms of this classification correspond to the DIPG tumors that represent a particular form of brainstem tumors not eligible for a surgical resection and characterized by their dismal prognosis.

All these classifications should be improved by including MRI and molecular analysis of the tumor in order to decide the best oncological treatment and assess their possible prognostic evolution. Indeed, to-date the main progress is represented by the bio-molecular and genetic knowledge of the Central Nervous System tumors. For brainstem tumors, two main oncogenic pathways have been individualized: the BRAF pathway that is usually associated with benign tumors and the histone pathway that characterizes malignant neoplasms. Diffuse midline glioma and DIPG are mostly characterized by the presence of the H3.3K27M or H3.1K27M mutation of the histone genes H3F3A and HIST1H3B respectively. The mutation inhibits the PCR2 complex and regulates gene expression via the global reduction in demethylation and trimethylation of lysine 27 residue of histone sub-unit (13, 14). For benign brainstem tumors, the identification of BRAF alterations permits to use new anti –BRAF drugs such as dabrafenid or vemurafenid or anti—MEK as trametinib or selumetinib. Previous studies have shown that gangliogliomas with BRAF-V 600E mutation have an increased risk for progression or recurrence especially in tumor located in the brainstem with a shorter progression free survival compared to BRAF wild-type gangliogliomas (15). Bio-molecular and genetic markers are even more important for DIPG in order to find new targeted drugs to improve the survival.

Castel et al. in a biomolecular study concerning 62 patients, showed the loss of H3K27 trimethylation in 95% of the DIPG samples (16). Beside the H3K27M mutation, H3.1 K27M and H3.3 K27I mutations were found in pontine tumors. An oligodendroglial differentiation was observed more frequently in H3.3K27M DIPG. The H3.1K27M mutation was associated with the presence of large areas of necrosis and a better response to radiotherapy with a better survival rate of 15 months compared to a survival of 9.2 months for H3.3 mutated patients. These findings are consistent with the literature (17).

In our experience the treatment with targeted chemotherapy did not significantly change the rate of survival for DIPG because the median survival was inferior to one year (10.7 months) in patients with and without the mutation. Considering only patients treated with the BIOMEDE program and patients treated with the TRONC 98, there was no significant difference in the median survival with a survival of 13.7 months for the group (BIOMEDE) and 13.5 months for the TRONC 98 group. We have to stress that, in our cohort, biomolecular analysis was available only in a limited number of patients. This prevented us to establish significant statistical differences regarding the survival as these technologies were available only from 2015 onward.

The necessity to include patients in targeted chemotherapy protocols was at the origin of trials encouraging to perform biopsies and we share this attitude.

The importance of the biopsy for brainstem tumors has been highlighted by the progress on molecular targets for many general cancers (18) and by the need of establishing a correct histopathological and molecular diagnosis. Indeed no less than 10% of patient with presumed brainstem gliomas had different post-biopsy diagnoses such as demyelinating disease, vascular lesions, infectious disease like rhombencephalitis etc (19). The second strong argument for biopsy is the inclusion of patients in new tailored protocols with new targets according to the genetic constitution of the tumor as the French Biomede study (20). However, to date, the stereotactic biopsy has been criticized because the sample was taken in a limited region and thus could not represent the entire volume of the tumor. Therefore, it was debatable to expose patients to the risk of surgery in such a critical anatomical structure (21). To avoid the possible clinical complications, post mortem biopsies have been proposed and performed (21). Analysis of post –mortem tissue and some surgical biopsies have suggested that identifiable genetic and molecular alterations can be found and may serve as therapeutics targets (22). However, post-mortem tissue samples could no reproduce valid bio-molecular tumor markers (23, 24).

If a biopsy has to be done, different surgical techniques can be used: direct approach, stereotactic technique with a frame or frameless, stereotactic biopsy using image guidance. We have switched from a frame-based to a frameless biopsy technique (Medtronic) without losing precision and without increasing the rate of complications. We have not observed significant statistical differences concerning the rate of sequels with the different techniques used except for a slight increase with the direct approach because it was associated to a more extended surgical removal. In the literature, the rate of transient and permanent deficits following biopsy varies from 4% to 1% with a rate of 94.9% of diagnostic yield (25), 4.9% of morbidity and 0.7% of mortality (26). Roujeau et al. reported a rate of 8% of complications without non-diagnostic biopsies (27). In a later paper, Puget raised the question if it was really worthy performing biopsies of brainstem lesions (28). Schumacher stressed that the diagnosis of brainstem tumors could be done with images criteria, laboratory data and clinical and laboratory data alone (27–29). Paugh underlined that with limited specimen it is not always possible to have a representative element of the tumor and also of its genetic constitution (27, 30).

In the future, it may be possible that the PET/MRI scans may help to establish a diagnosis of these tumors with the study of their own metabolism avoiding the need of a surgical biopsy.

Our rate of post-surgical complications for biopsies for brain stem tumors was 22%. This higher rate of complications could be explained with the higher number of biopsies realized with a direct open approach with an attempt to remove a volume of tumor as large as possible avoiding clinical sequelae.

The other reason of our high rate of complications may be due to the fact that we reported also transitory complications as cardiac rhythm troubles and respiratory failures during the procedures that were completely reversible; if we remove these short-lived complications our rate of post-biopsy complications goes down to 6.5% as in other series. Regarding the mortality rate, our 2.2% rate confirm that the location of this tumor exposes the patients to a real risk.

In the future, liquid biopsy and liquid biome can take a great importance not only to establish the diagnosis but also to have an evaluation of treatments in the follow-up of the disease. Liquid biopsy is a developing technique in cancer studies using saliva, blood, urine, CSF to detect cells free tumors DNA (Ct DNA), circulating tumor cells (CTC) and tumor extracellular vesicles (31, 32). CSF –derivates have better sensibility compared with circulating tumor cells (33). In 2017, Huang could show Sanger sequencing and mutation—specific PCR to detect H3 mutations in CSF- derivate tumor DNA (34). However, these techniques need highly specialized laboratories. The main limitations are the calculation of allele frequency and the lack of sensitivity. However, this could in the future guarantee a precise histological and genetic diagnosis representing a real alternative to the surgical biopsy.

### Surgical considerations

The first direct surgical approaches for a brainstem tumor were reported in the 1960s (35). Then, Hoffman and Epstein reported in the 80s their experience with the exophytic forms located at level of the bulbo-medullary region and at level of the quadrigeminal plate (21, 36, 37). Bricolo et al. also described some entry points to approach brainstem tumors reducing the risk of sequelae for patients and increasing the rate of surgical resection (38). Generally respecting these well definite entry zones help to reduce sequels. However, for us, it is important to approach the tumor either where it is visible at level of the anatomical surface or choising the shorter distance to join the tumor with short opening incision trying to respect the anatomical structures.

Progresses of surgical techniques were useful to remove focalized brainstem tumors, bulbo-medullary and latero-bulbar tumors or exophytic mesencephalic lesions that could benefit from a large resection. The use of neurovavigation, intra-operative neurophysiology and ultrasonic aspirator have also permitted a better surgical outcome. Nevertheless, the surgical approach has to be chosen based on the anatomical localization of the tumor, its exophytic extension and the axis of the tumor’s growth. In our center, we generally use the sitting position except in cases of subtemporal approach to expose the lateral portion of the mesencephalic region or the lateral portion of the pontine region. The Mayfield headrest is used in patient older than three years old to avoid complication with the pins penetrating into the fragile bone of younger patients. The CUSA cavitron permits to remove tumors staying inside the tumor and with a weak power of aspiration and fragmentation it is possible to reduce the tumoral mass also on critical area as the floor of the fourth ventricle. We advocate an aggressive surgery for benign tumors such as pilocytic astrocytomas and ganglioglioma even if complete surgical removal was possible in only twenty patients of our cohort. A complete removal can be possible also at the level of the bulbo-medullary junction. A subtotal surgical resection was possible in 13 patients. In case of ganglioglioma of the latero-bulbar or latero bulbo-pontine region, a large removal can favor a quiescence of the tumor with a good evolution also for long time. For exophytic tectal plate tumors, we prefer the sub-occipital trans-tentorial approach, as for pineal tumors, that permits a very large exposure of the posterior mesencephalic region. We already reported our experience with tectal plate tumors (39). When tumors are benign the clinical results are satisfying and the surgical mortality is nihil as long as we follow a safe surgical strategy (40). The results of exophytic brain stem tumors are reported in the Tables 1–3. The indication of surgery for tectal plate tumors was relate to their volumetric progression, and to the fact that they were exophytic and associated with a documented clinical evolution. The rate of complication in 42 patients operated for exophytic or localized benign gliomas with a direct approach was 14% and involved principally the cranial nerves with mild swallowing troubles in a patient, a peripheral facial palsy in three patients, gait troubles in a child and a cerebellar syndrome in another case. A pseudo meningocele disappeared after treatment with furosemide and in two cases a skin infection was treated with antibiotics. One patient died of a cardio-respiratory complication one year after tumor removal (pilocytic astrocytoma) at level of the bulbo-medullary junction. Our experience confirmed that surgery of the brainstem tumors is possible as reported by others authors (21, 41). The respect of safe entry zone already described by different authors permits a more aggressive surgery with a low rate of sequelae and an acceptable mortality rate (6, 42, 43). Surgical removal of benign tumors, even with partial surgical resection can be able to stop their evolution for a long time.

On the other hand, surgery has limited possibilities for DIPG. We believe that in the future new radiological tools could allow a diagnosis without surgical procedures and also that the individualization of tumoral targets with tailored chemotherapies and radiotherapy could help to improve the prognosis of these tumors. The bio-molecular studies and tailored chemotherapic treatment in our experience did not permit an improved survival rate of patients with diffuse brainstem gliomas.

## Conclusions

Our series confirms that histologically benign tumors have a good survival with surgical removal and adjuvant treatment. DIPG still have a dismal prognosis despite recent biomolecular characterizations and new chemotherapeutic regimens that have not been able to significantly improve their survival rate and the quality of life. Still, we believe that it is necessary to perform biopsies to obtain tissue for diagnosis and to perform molecular studies to improve the efficacy of adjuvant treatment. A better classification of brainstem tumors in children with the bio-molecular understanding of the oncogenic process will be in the future responsible for further improvement of more personalized treatment.

The future depends mainly on the discovery of more effective drugs that could change the dismal evolution of infiltrating brainstem tumors. The better overall prognosis of exophytic lesions may be due to the fact that these lesions are less aggressive and consequently more accessible to an extensive surgical resection that needs to be balanced with morbidity prevention and with the ability to maintain z good quality of life.

## Data Availability

The raw data supporting the conclusions of this article will be made available by the authors, without undue reservation.

## References

[1] Kummel B. *Beitrag Zur Kasuistik Der Gliom Des Pons Und Der Medulla Oblongata*. Klin Medizin. (1881).

[2] Monakow C. Histoire naturelle des tumeurs cérébrales, en particulier du gliome. Encéphale. (1926):117–89.

[3] Bailey P Cushing H. A classification of tumors of the glioma group on a hystogenesis basis. Philadelphia: Lippincot (1926).

[4] Guegan Y Guy G Guegan Y. Les lésions chirurgicales du tronc cérébral. Rapport de la société de neurochirurgie de langue française. Neurochirurgie. (1989).2666867

[5] Stroink AR Hoffman HJ Hendrick EB Humphreys RP. Diagnosis and management of pediatric brain-stem gliomas. J Neurosurg. (1986) 65:745–50. 10.3171/jns.1986.65.6.0745 3772471

[6] Klimo P Nesvick CL Broniscer A Orr BA Choudhri AF. Malignant brainstem tumors in children, excluding diffuse intrinsic pontine gliomas. J Neurosurg Pediatr. (2016) 17:57–65. 10.3171/2015.6.PEDS15166 26474099

[7] Hossain MJ Xiao W Tayeb M Khan S. Epidemiology and prognostic factors of pediatric brain tumor survival in the US: evidence from four decades of population data. Cancer Epidemiol. (2021) 72:101942. 10.1016/j.canep.2021.101942 33946020 PMC8142618

[8] Sun T Xu Y Pan C Liu Y Tian Y Li C Surgical treatment and prognosis of focal brainstem gliomas in children: a 7 year single center experience. Medicine (Baltimore). (2020) 99:e22029. 10.1097/MD.0000000000022029 32899058 PMC7478697

[9] Joud A Stella I Klein O. Diffuse infiltrative pontine glioma biopsy in children with neuronavigation, frameless procedure: a single center experience of 10 cases. Neurochirurgie. (2020) 66:345–8. 10.1016/j.neuchi.2020.05.007 32860812

[10] Guillain G Bertrand I Gruner J. *Les gliomes infiltrés du tronc cérébral*. Masson. (1945).

[11] Ingraham FD Matson DD. *Neurosurgery of infancy*. (1954).

[12] Choux M Lena G Do L. Brain stem tumors. In: Choux M Di Rocco C Hockley A, editors. Pediatric neurosurgery. New York: Churchill Livingstone (200). p. 471–91.

[13] Lewis PW Müller MM Koletsky MS Cordero F Lin S Banaszynski LA Inhibition of PRC2 activity by a gain-of-function H3 mutation found in pediatric glioblastoma. Science. (2013) 340:857–61. 10.1126/science.1232245 23539183 PMC3951439

[14] Rashed WM Maher E Adel M Saber O Zaghloul MS. Pediatric diffuse intrinsic pontine glioma: where do we stand? Cancer Metastasis Rev. (2019) 38:759–70. 10.1007/s10555-019-09824-2 31802357

[15] Dahiya S Haydon DH Alvarado D Gurnett CA Gutmann DH Leonard JR. BRAFV600E Mutation is a negative prognosticator in pediatric ganglioglioma. Acta Neuropathol. (2013) 125:901–10. 10.1007/s00401-013-1120-y 23609006

[16] Castel D Philippe C Calmon R Le Dret L Truffaux N Boddaert N Histone H3F3A and HIST1H3B K27M mutations define two subgroups of diffuse intrinsic pontine gliomas with different prognosis and phenotypes. Acta Neuropathol. (2015) 130:815–27. 10.1007/s00401-015-1478-0 26399631 PMC4654747

[17] Wu G Diaz AK Paugh BS Rankin SL Ju B Li Y The genomic landscape of diffuse intrinsic pontine glioma and pediatric non-brainstem high-grade glioma. Nat Genet. (2014) 46:444–50. 10.1038/ng.2938 24705251 PMC4056452

[18] Warren KE. Diffuse intrinsic pontine glioma: poised for progress. Front Oncol. (2012) 2:205. 10.3389/fonc.2012.00205 23293772 PMC3531714

[19] Dellaretti M Touzet G Reyns N Dubois F Gusmão S Pereira JLB Correlation among magnetic resonance imaging findings, prognostic factors for survival, and histological diagnosis of intrinsic brainstem lesions in children. J Neurosurg Pediatr. (2011) 8:539–43. 10.3171/2011.9.PEDS1167 22132909

[20] Grill J. Biological medicine for diffuse intrinsic pontine gliomas eradication (BIOMEDE): results of the three-arm biomarker-driven randomized trial in the first 230 patients from Europe and Australia.

[21] Epstein F Wisoff JH. Surgical management of brain stem tumors of childhood and adolescence. Neurosurg Clin N Am. (1990) 1:111–21. 10.1016/S1042-3680(18)30827-1 2135963

[22] Angelini P Hawkins C Laperriere N Bouffet E Bartels U. Post mortem examinations in diffuse intrinsic pontine glioma: challenges and chances. J Neurooncol. (2011) 101:75–81. 10.1007/s11060-010-0224-7 20473723

[23] Robison NJ Kieran MW. Diffuse intrinsic pontine glioma: a reassessment. J Neurooncol. (2014) 119:7–15. 10.1007/s11060-014-1448-8 24792486

[24] Taylor KR Mackay A Truffaux N Butterfield Y Morozova O Philippe C Recurrent activating ACVR1 mutations in diffuse intrinsic pontine glioma. Nat Genet. (2014) 46:457–61. 10.1038/ng.2925 24705252 PMC4018681

[25] Samadani U Judy KD. Stereotactic brainstem biopsy is indicated for the diagnosis of a vast array of brainstem pathology. Stereotact Funct Neurosurg. (2003) 81:5–9. 10.1159/000075097 14742957

[26] Pincus DW Richter EO Yachnis AT Bennett J Bhatti MT Smith A. Brainstem stereotactic biopsy sampling in children. J Neurosurg. (2006) 104:108–14. 10.3171/ped.2006.104.2.108 16506498

[27] Roujeau T Machado G Garnett MR Miquel C Puget S Geoerger B Stereotactic biopsy of diffuse pontine lesions in children. J Neurosurg Pediatr. (2007) 107:1–4. 10.3171/PED-07/07/001 17647306

[28] Puget S Beccaria K Blauwblomme T Roujeau T James S Grill J Biopsy in a series of 130 pediatric diffuse intrinsic pontine gliomas. Childs Nerv Syst. (2015) 31:1773–80. 10.1007/s00381-015-2832-1 26351229

[29] Schumacher M Schulte-Mönting J Stoeter P Warmuth-Metz M Solymosi L. Magnetic resonance imaging compared with biopsy in the diagnosis of brainstem diseases of childhood: a multicenter review. J Neurosurg Pediatr. (2007) 106:111–9. 10.3171/ped.2007.106.2.111 17330536

[30] Paugh BS Broniscer A Qu C Miller CP Zhang J Tatevossian RG Genome-wide analyses identify recurrent amplifications of receptor tyrosine kinases and cell-cycle regulatory genes in diffuse intrinsic pontine glioma. J Clin Oncol. (2011) 29:3999–4006. 10.1200/JCO.2011.35.5677 21931021 PMC3209696

[31] Caretti V Jansen MHA Vuurden Dv Lagerweij T Bugiani M Horsman I Implementation of a multi-institutional diffuse intrinsic pontine glioma autopsy protocol and characterization of a primary cell culture. Neuropathol Appl Neurobiol. (2013) 39:426–36. 10.1111/j.1365-2990.2012.01294.x 22845849

[32] Alix-Panabières C Pantel K. Clinical applications of circulating tumor cells and circulating tumor DNA as liquid biopsy. Cancer Discov. (2016) 6:479–91. 10.1158/2159-8290.CD-15-1483 26969689

[33] Pan C Diplas BH Chen X Wu Y Xiao X Jiang L Molecular profiling of tumors of the brainstem by sequencing of CSF-derived circulating tumor DNA. Acta Neuropathol. (2019) 137:297–306. 10.1007/s00401-018-1936-6 30460397 PMC7523750

[34] Huang TY Piunti A Lulla RR Qi J Horbinski CM Tomita T Detection of histone H3 mutations in cerebrospinal fluid-derived tumor DNA from children with diffuse midline glioma. Acta Neuropathol Commun. (2017) 5:28. 10.1186/s40478-017-0436-6 28416018 PMC5392913

[35] Alvisi C Cerisoli M Maccheroni ME. Long-term results of surgically treated brainstem gliomas. Acta Neurochir (Wien). (1985) 76:12–7. 10.1007/BF01403823 4003123

[36] Epstein F Constantini S. Practical decisions in the treatment of pediatric brain stem tumors. Pediatr Neurosurg. (1996) 24:24–34. 10.1159/000121011 8817612

[37] Vandertop WP Hoffman HJ Drake JM Humphreys RP Rutka JT Amstrong DC Focal midbrain tumors in children. Neurosurgery. (1992) 31:186–94. 10.1227/00006123-199208000-00003 1308661

[38] Bricolo A Turazzi S. Surgery for gliomas and other mass lesions of the brainstem. Adv Tech Stand Neurosurg. (1995) 22:261–341. 10.1007/978-3-7091-6898-1_5 7495420

[39] Mottolese C Szathmari A Beuriat PA Frappaz D Jouvet A Hermier M. Tectal plate tumours. Our experience with a paediatric surgical series. Neurochirurgie. (2015) 61:193–200. 10.1016/j.neuchi.2013.12.007 25441707

[40] Lapras C Bognar L Turjman F Villanyi E Mottolese C Fischer C Tectal plate gliomas. Part I: microsurgery of the tectal plate gliomas. Acta Neurochir (Wien). (1994) 126:76–83. 10.1007/BF01476414 8042559

[41] Cavalheiro S Yagmurlu K da Costa MDS Nicácio JM Rodrigues TP Chaddad-Neto F Surgical approaches for brainstem tumors in pediatric patients. Childs Nerv Syst. (2015) 31:1815–40. 10.1007/s00381-015-2799-y 26351233 PMC4564452

[42] Ogata N Yonekawa Y. Paramedian supracerebellar approach to the upper brain stem and peduncular lesions. Neurosurgery. (1997) 40:101–4; discussion 104–105. 10.1097/00006123-199701000-00023 8971831

[43] Kyoshima K Kobayashi S Gibo H Kuroyanagi T. A study of safe entry zones via the floor of the fourth ventricle for brain-stem lesions. Report of three cases. J Neurosurg. (1993) 78:987–93. 10.3171/jns.1993.78.6.0987 8487085

